# Fructus Sophorae Extract Generated Using the Natural Deep Eutectic Solvent ChCl Urea Activates Wnt/β Catenin and cAMP/MITF Signaling to Stimulate Hair Growth and Pigmentation

**DOI:** 10.3390/ijms27198781

**Published:** 2026-09-30

**Authors:** Gary Ka-Wing Yuen, Yinzhi Li, Tina Ting-Xia Dong, Karl Wah-Keung Tsim

**Affiliations:** 1 Division of Life Science, Centre for Chinese Medicine, and State Key Laboratory of Molecular Neuroscience, The Hong Kong University of Science and Technology, Hong Kong, China; kwgyuen@connect.ust.hk (G.K.-W.Y.); yliwt@connect.ust.hk (Y.L.);; 2 Shenzhen Key Laboratory of Edible and Medicinal Bioresources, HKUST Shenzhen Research Institute, Shenzhen 518057, China

**Keywords:** *Sophora japonica*, sophoricoside, alopecia, hair growth, natural deep eutectic solvents

## Abstract

Androgenetic alopecia remains challenging to treat, with limited FDA-approved therapies. Natural products, such as the fruit of *Sophora japonica* L. (Fructus Sophorae), could be alternatives. This study developed a green extraction method using natural deep eutectic solvents (NADESs) coupled with ultrasonic assistance to recover active phytochemicals from Fructus Sophorae and evaluated the extract for promoting hair growth and melanogenic activities. Among ten NADES formulations, ChCl-Urea (1:2) with 40% water content, named CU-40, generated the highest yield of sophoricoside (8.79 mg/g), surpassing that of 70% ethanol by ~25%. In dermal papilla cells, this extract activated Wnt/β-catenin signaling, upregulated downstream targets, such as β-catenin, AXIN2, LEF-1, ALP, and IGF-1, and induced GSK3β phosphorylation. In B16 melanoma, the extract of Fructus Sophorae stimulated cAMP signaling, enhanced MITF promoter activity, and increased melanin production. In an ex vivo mouse vibrissae hair follicle culture, the extract promoted mouse vibrissae hair follicle growth. These findings establish CU-40 as a sustainable, efficient NADES solvent for Fructus Sophorae that yields a bioactive extract promoting hair growth and pigmentation via the Wnt/β-catenin and cAMP/MITF pathways.

## 1. Introduction

Androgenetic alopecia is a prevalent condition that generally manifests in individuals during their 30s and 40s [1]. The prevalence varies greatly among different racial and ethnic groups. Approximately 30% of Caucasian males have hair loss in their 30s, with this figure increasing to over 40% by the age of 50. Conversely, the rate tends to be lower in groups from Asia and Africa. About 19.9% of the Chinese population exhibited androgenetic alopecia [2]. The US FDA has approved only two therapies for androgenetic alopecia to date: topical minoxidil and oral finasteride [3]. Each of them produces adverse effects. For example, minoxidil induces skin irritation or rashes, and finasteride results in sexual dysfunction [4,5,6].

Natural products are alternatives for therapeutic usage due to their accessibility, safety profile, and cost-effectiveness relative to synthetic options. These natural compounds have a wide range of therapeutic capabilities, including anticancer, antioxidant, anti-inflammatory, and neuroprotective effects [7,8,9,10]. Among these products, the fruits of *Sophora japonica* L. (also known as *Styphnolobium japonicum*; Fructus Sophorae), exhibit a broad range of pharmacological activities, including the promotion of cardiovascular health, anti-inflammatory properties, antiosteoporotic effects, antioxidant protection, antitumor potential, antibacterial and antiviral activities, facilitation of hemostasis, and inhibition of atherosclerosis [7,11,12]. In addition, the phytochemical sophoricoside has been shown to be responsible for the hair growth-promoting property of Fructus Sophorae [13].

To isolate plant-derived metabolites, conventional methods have mainly relied on organic solvents, such as methanol, ethanol, ethyl acetate, and acetone [14,15]. Because of their intrinsic toxicity, they must be completely removed from the extracts using energy-intensive evaporation processes that eventually lengthen processing times and raise costs [16]. Research has therefore shifted toward using sustainable, greener solvent systems that could reduce ecological footprints while maintaining or improving extraction performance [17]. Natural deep eutectic solvents (NADESs) are an exceptional alternative. They are biocompatible mixtures made by precisely mixing naturally occurring hydrogen bond acceptors (like choline chloride) and donors (like urea, organic acids, or sugars), which produce liquids with exceptionally low melting points even though they are solids on their own [18,19]. NADESs are notable for their low toxicity, superior biodegradability, affordability, and capacity to dissolve a wide variety of polar and semi-polar bioactive compounds [20]. In comparison to traditional solvents, NADESs frequently produce extracts with increased biological activities, better extraction efficiencies, and greater solubility for target molecules [21].

Here, we aimed to employ a NADES in extracting bioactive phytochemicals from Fructus Sophorae, particularly in combination with ultrasonic-assisted extraction. When targeting the yield of sophoricoside, ChCl-Urea (1:2) with 40% water (CU-40) was shown to generate the highest yield of sophoricoside. In addition, the functionalities of Fructus Sophorae extract using NADES were further illustrated here by in vitro assays, as well as mouse vibrissae hair follicle, in supporting hair growth and pigmentation.

## 2. Results

**Figure 1:**
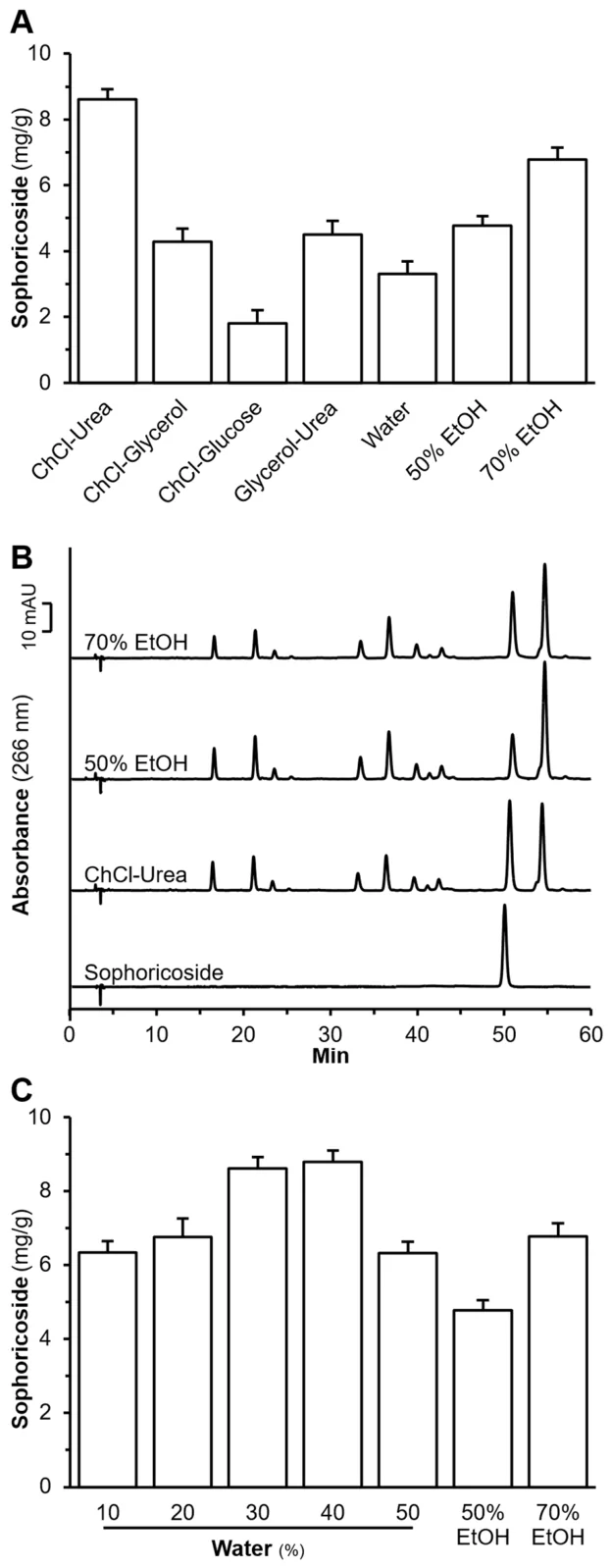
Screening and optimization of NADES-UAE for sophoricoside extraction. (**A**) Sophoricoside yields of four NADES formulations compared with conventional solvents (water, 50% EtOH, 70% EtOH). (**B**) HPLC chromatographic fingerprints of the extracts obtained with 70% ethanol, 50% ethanol, and ChCl-Urea (1:2) NADES. The injection volume was 10 μL at 10 mg/mL. The peaks were detected at a wavelength of 266 nm. The chemical marker utilized here was sophoricoside at a concentration of 20 mg/L (*n* = 3). (**C**) Effect of water content (10–50%) in ChCl-Urea (1:2) on the extraction yield of sophoricoside. Data are presented as mean ± SEM, *n* = 3.

Ten types of NADES mixtures with 30% (*v*/*v*) water were used to extract phytochemicals from Fructus Sophorae (Supplementary Figure S1). The extraction efficiency of the different NADESs was evaluated by determining the content of sophoricoside within each extract using HPLC. The calibration curve for sophoricoside showed excellent linearity, with a correlation coefficient of *R*^2^ = 0.9996. The linear equation of the calibration curve was y = 17.436x − 7.9554, where x represents the concentration of sophoricoside, and y represents the corresponding peak area. Among the tested NADES solvents, the top four performers, together with water, 50% ethanol, and 70% ethanol extracts, are shown (Figure 1A). The sophoricoside obtained was 8.6 mg/g with ChCl-Urea (1:2), 4.3 mg/g with ChCl-Glycerol (1:2), 4.5 mg/g with Glycerol-Urea (1:1), and 1.8 mg/g with ChCl-Glucose (1:1). In comparison, the contents of sophoricoside extracted using conventional solvents were 3.3 mg/g with water, 4.77 mg/g with 50% ethanol, and 6.78 mg/g with 70% ethanol. The extractive efficiency of sophoricoside using ChCl-Urea (1:2) was approximately 25% higher than that of 70% ethanol extraction (Figure 1A). HPLC fingerprinting further revealed the chemical profiles and sophoricoside amounts in the extracts obtained with 70% ethanol, 50% ethanol, and ChCl-Urea NADES (Figure 1B). Due to its superior extraction performance, ChCl-Urea (1:2) was selected for further analysis.

The water content exerts a substantial effect on the extraction efficiency of NADES for phytochemicals by modulating their polarity, lowering viscosity, and enhancing mass transfer. With increasing water content in the NADES, the extraction efficiency of sophoricoside exhibited an initial increase followed by a subsequent decline. The contents of sophoricoside extracted by 10% to 50% water content of ChCl-Urea (1:2) were 6.34 mg/g, 6.77 mg/g, 8.62 mg/g, 8.79 mg/g, and 6.32 mg/g, respectively (Figure 1C). Here, the extract generated from 40% water content in ChCl-Urea (1:2), named CU-40, was found to provide the highest sophoricoside extraction yield.

**Figure 2:**
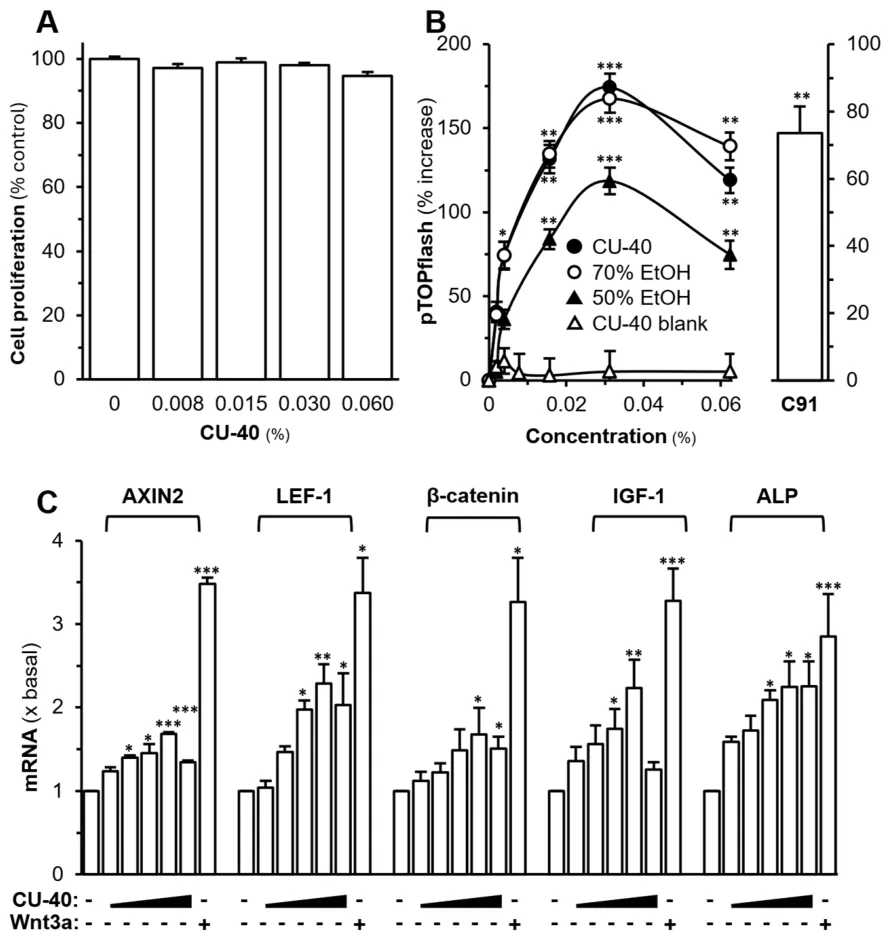
CU-40 activates Wnt/β-catenin signaling in DPCs. (**A**) Cell viability of DPCs treated with increasing concentrations of CU-40 for 24 h, assessed by MTT assay. (**B**) The pTOPflash-transfected DPCs treated with various doses of extracts (as indicated) and C91 (1 µM). Cell lysates were collected for luciferase assay. (**C**) RT-qPCR analysis of downstream Wnt target genes in DPCs treated with CU-40 (0.002%, 0.004%, 0.015%, 0.03%, and 0.06%) or Wnt3a (200 ng/mL) for 48 h. The expression levels of AXIN2, LEF-1, β-catenin, IGF-1, and ALP mRNA are shown. Data are normalized and expressed as % of control, fold of change (x basal), or % of increase, in mean ± SEM, *n* = 4. * *p* < 0.05, ** *p* < 0.01, *** *p* < 0.001.

To elucidate the involvement of CU-40 in the activation of hair growth-associated signaling cascades, DPCs were exposed to various concentrations of CU-40 for a duration of 24 h. Cell viability was subsequently assessed using an MTT assay. At concentrations up to 0.06%, CU-40 did not induce any significant changes in DPC viability (Figure 2A). To evaluate Wnt/β-catenin signaling activation, a pTOPflash reporter construct with the TCF-binding consensus sequence specific for the β-catenin–TCF complex was employed, and the promoter-driven luciferase activity was quantified [22]. In pTOPflash-transfected DPCs, treatment with CU-40 resulted in an approximately 170% increase in luciferase activity in a dose-dependent manner, with maximal induction observed at a concentration of approximately 0.03% (Figure 2B), and 70% ethanol showed a similar response; however, the 50% ethanol extract showed much lower efficacy. The positive control CHIR99021 (C91) was a selective GSK3β inhibitor that increased Wnt signaling by over 70% (Figure 2B).

**Figure 3:**
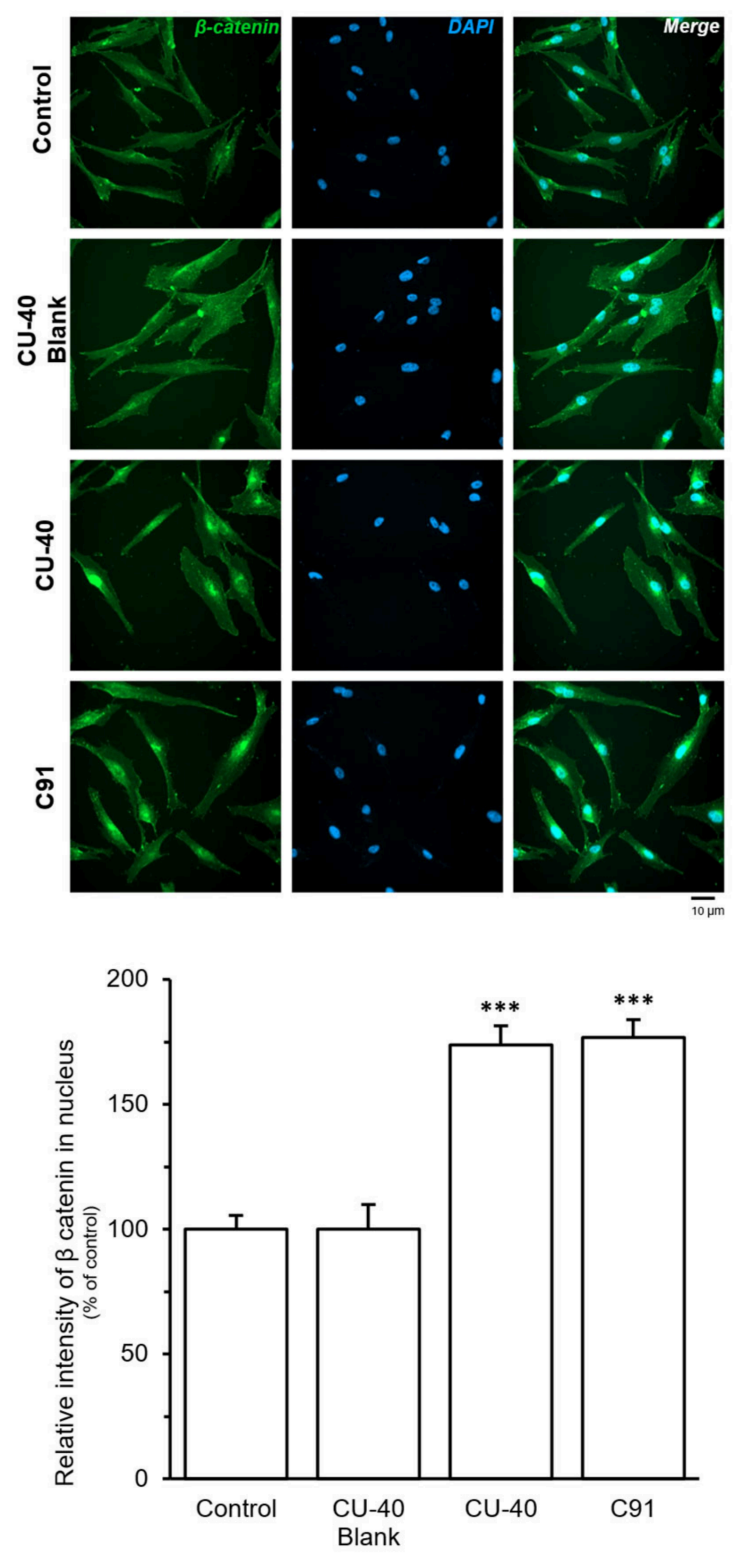
Immunofluorescence analysis of ß-catenin localization in DPCs. Immunofluorescence analysis (**upper panel**) of ß-catenin localization in DPCs treated with CU-40 Blank (0.03%), CU-40 (0.03%), or C91 (1 μM). Data are normalized and expressed as % of control (**lower panel**), in mean ± SEM, *n* = 4. *** *p* < 0.001.

To evaluate the impact of CU-40 on Wnt/β-catenin signaling, RT-PCR was performed on cultured DPCs following a 48 h treatment. The expression levels of several downstream targets of Wnt signaling, e.g., AXIN2, LEF-1, β-catenin, IGF-1, and ALP, were measured. Treatment with CU-40 induced a dose-dependent upregulation of AXIN2, LEF-1, and β-catenin mRNAs (Figure 2C). Additionally, the extract significantly increased the expression of the growth biomarker of DPCs, i.e., ALP. Notably, the treatment resulted in about a 2-fold increase in the mRNA level of IGF-1, a key growth factor in promoting hair follicle (Figure 2C). Here, Wnt3a was utilized as a positive control to confirm the activation of the Wnt/β-catenin signaling cascade, i.e., induction of gene expression in all cases. Moreover, treatment with CU-40 and C91 (a positive control) increased the localization of β-catenin to the nucleus by ~175% in the β-catenin immunofluorescence experiment (Figure 3).

**Figure 4:**
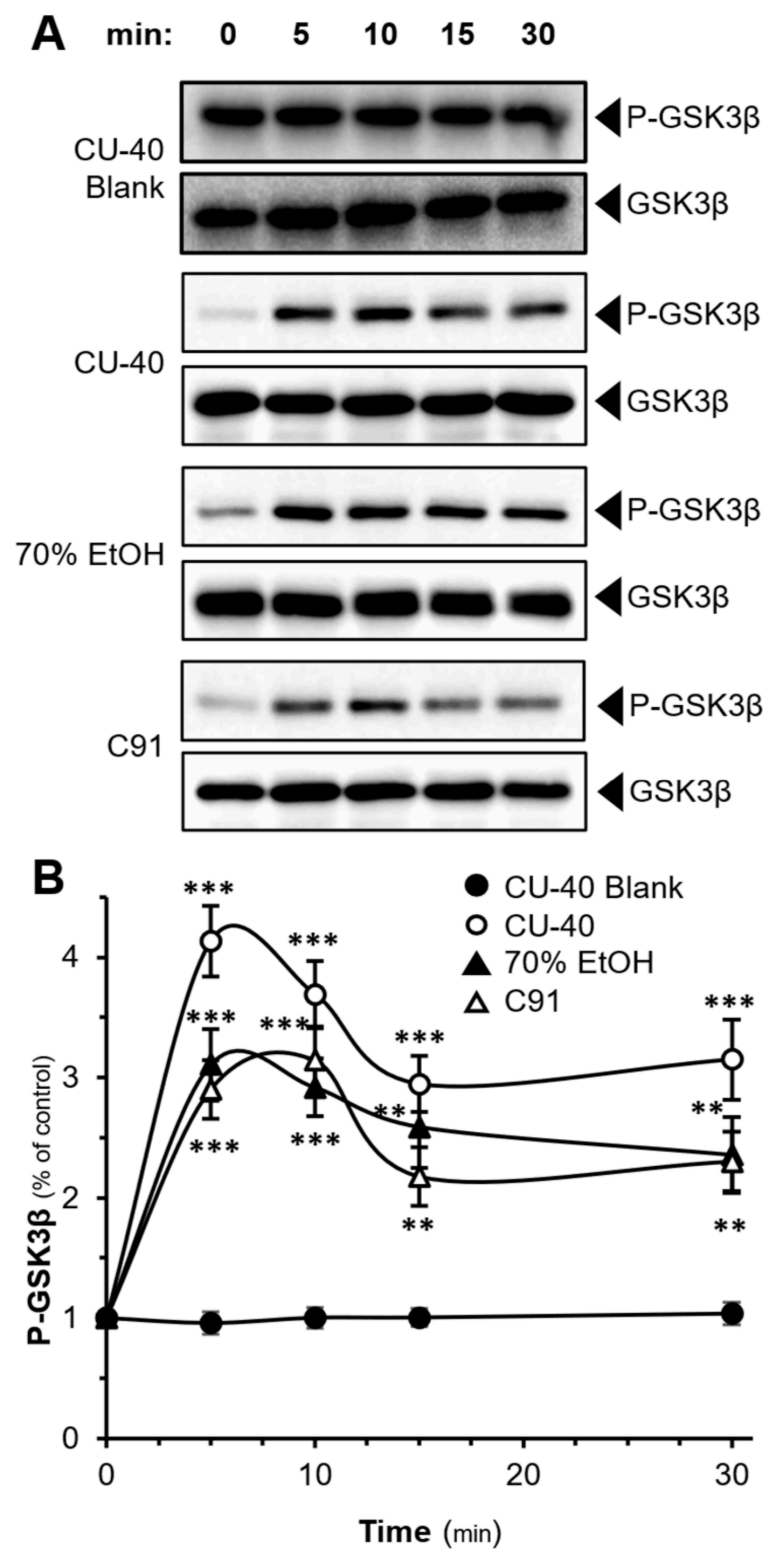
CU-40 phosphorylates GSK3β in DPC cultures. (**A**) Representative Western blot images showing phosphorylated GSK3β (P-GSK3β) and total GSK3β levels in DPCs treated with CU-40 blank (0.03%), CU-40 (0.03%), 70% ethanol extract (0.03%), or 1 µM CHIR99021 (C91) for the indicated durations. (**B**) Quantitative analysis of P-GSK3β/total GSK3β ratios. Data are normalized and expressed as % of control, in mean ± SEM, *n* = 3. ** *p* < 0.01, *** *p* < 0.001.

To investigate the mechanistic basis of Wnt/β-catenin pathway activation, GSK3β was examined owing to its pivotal role in the negative regulation of Wnt signaling. GSK3β phosphorylates β-catenin, thereby promoting its destabilization and subsequent degradation, which maintains low basal levels of β-catenin within the cytosol and nucleus. Treatment of DPCs with either CU-40 or C91 resulted in a transient induction of GSK3β phosphorylation, observed as early as 5 min following incubation (Figure 4).

**Figure 5:**
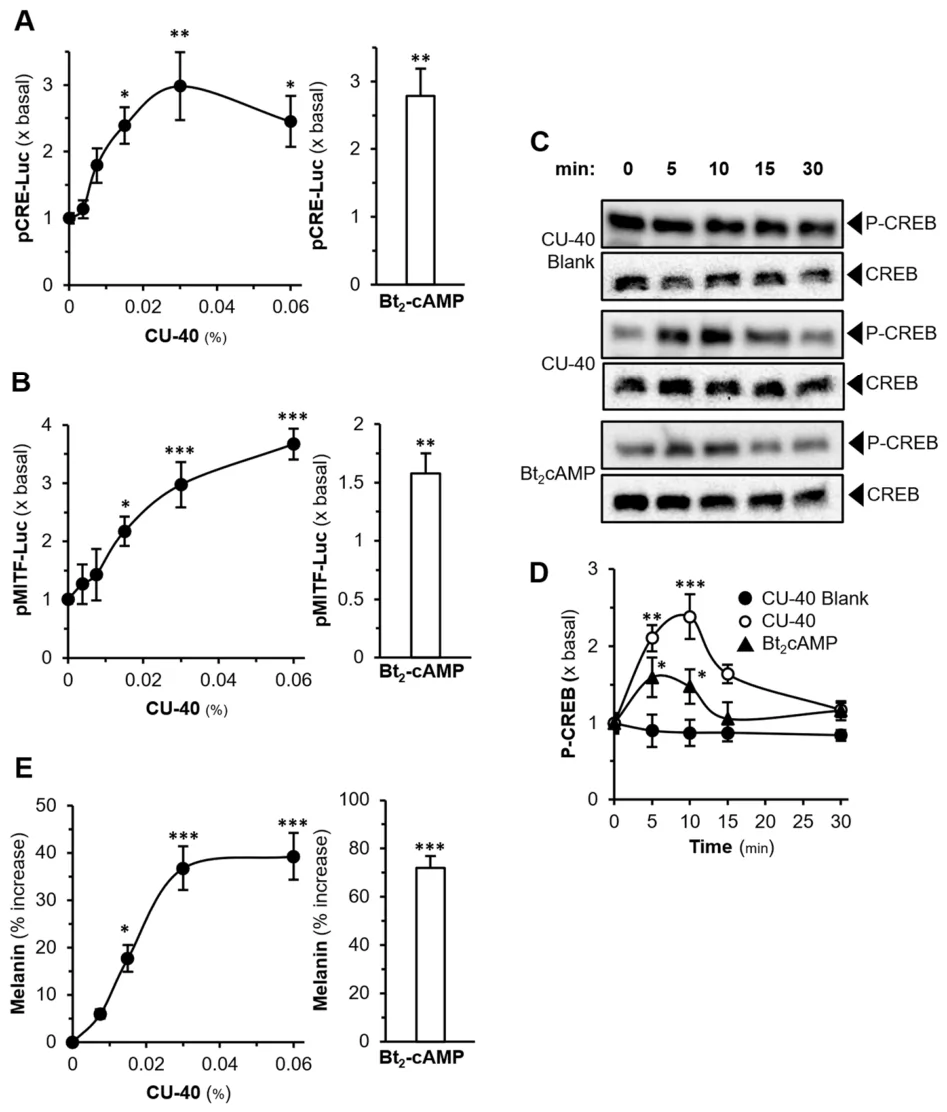
CU-40 promotes cAMP/MITF-dependent melanogenesis in cultured B16F10 cells. (**A**) Luciferase activity assay of pCRE-Luc-transfected B16F10 cells treated with different concentrations of CU-40 (as indicated). (**B**) The luciferase activity assay of pMITF-Luc-transfected B16F10 cells, as in (**A**). (**C**) Representative Western blot images showing phosphorylated CREB (P-CREB) and total CREB levels in B16F10 cells treated with CU-40 blank (0.03%), CU-40 (0.03%), or 1 mM Bt_2_-cAMP for the indicated durations. (**D**) Quantitative analysis of P-CREB/total CREB ratios. (**E**) The melanin content in the conditioned medium was measured by spectrophotometry at 405 nm and normalized to total protein in B16F10 cultures. Data are normalized and expressed as fold change (x basal) or % of increase over control, in mean ± SEM, *n* = 4. * *p* < 0.05, ** *p* < 0.01, *** *p* < 0.001.

To evaluate the potential of CU-40 to enhance melanogenesis during hair growth, B16F10 melanoma cells were used as a cell model. cAMP signaling plays a central and well-established role in regulating melanogenesis, particularly the production of melanin [23]. The activation of protein kinase A and CREB, triggered by cAMP, lead to transcription of melanogenic genes and subsequent melanin synthesis and accumulation. The pCRE-Luc reporter construct, containing multiple cAMP response elements upstream of the luciferase gene, directly measures this cAMP signaling activation leading to melanin production. Cultured B16F10 cells were transfected with the pCRE-Luc reporter DNA construct. CU-40 was able to activate cAMP signaling in the pCRE-Luc-expressing cells, which increased the luciferase signal ~3-fold in a dose-dependent manner (Figure 5A). Bt_2_-cAMP acts as the positive control by inducing ~3-fold activation. MITF is the master transcription factor that directly drives expression of melanogenic enzymes responsible for melanin synthesis in melanocytes. The transcriptional activity of the MITF promoter was assessed using the pMITF-Luc reporter DNA construct in cultured B16F10 cells. CU-40 enhanced MITF promoter activity, as assessed using an MITF-Luc reporter, increasing the luciferase signal in a dose-dependent manner (Figure 5B). The positive control was 1 mM Bt_2_cAMP. Moreover, CU-40 induced CREB phosphorylation at 5 min by approximately 2.5-fold, while Bt_2_-cAMP induced it by approximately 1.5-fold, validating the positive control (Figure 5C,D). Importantly, the CU-40 blank did not induce CREB phosphorylation, confirming that the effect is attributable to Fructus Sophorae extract rather than the NADES components.

In order to confirm that the upstream cAMP signaling changes result in actual melanin production, extracellular melanin content in the culture medium of B16F10 cultures was quantified by spectrophotometry at 405 nm. CU-40 was able to increase melanin content in a dose-dependent manner by ~40% (Figure 5E). These activations of promoters and melanin content in B16F10 cultures were consistent even in the co-cultures with DPCs (Supplementary Figure S2).

**Figure 6:**
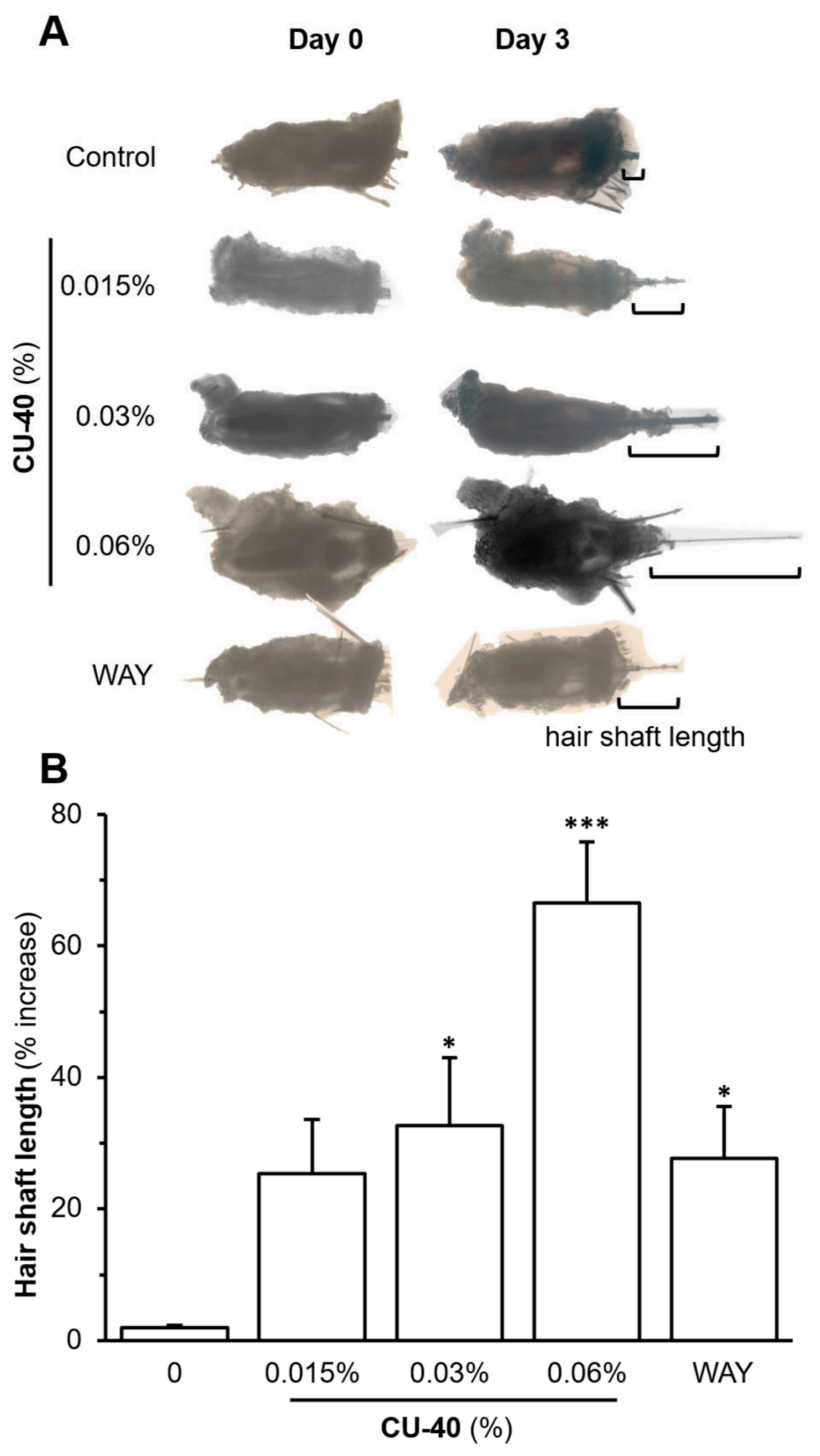
CU-40 promotes hair follicle elongation ex vivo. (**A**) Representative images of individual vibrissae in the anagen phase treated with different doses of CU-40 (as indicated) or WAY 316606 (1 μM), taken over 3 days. (**B**) Quantification of hair shaft length. Data are normalized and expressed as % of increase over control, in mean ± SEM, *n* = 4. * *p* < 0.05, *** *p* < 0.001.

Vibrissae hair follicles from mice are widely used as an ex vivo model for evaluating hair growth. To assess the hair growth-promoting potential of CU-40, isolated vibrissae hair follicles were treated with varying doses of CU-40 for 72 h (Figure 6A). Hair length was measured during this period, revealing a significant dose-dependent increase in growth compared to the control follicles. Notably, in response to treatment with CU-40 for 3 days, the hair growth exceeded 60% as compared to control follicles (Figure 6), consistent with the findings from the cell culture studies. As a positive control, 1 μM WAY 316606, an inhibitor of secreted frizzled-related protein-1 (sFRP-1) that prevents sFRP-1 from interacting with Wnt, induced an increase in hair length of approximately 25%.

## 3. Discussion

The present study demonstrates that choline chloride–urea (1:2) natural deep eutectic solvent supplemented with 40% (*v*/*v*) water, i.e., CU-40, constitutes an effective and environmentally sustainable medium for extracting sophoricoside from Fructus Sophorae, a traditional Chinese medicine resource renowned for its rich isoflavone glycoside content. We have shown that, under optimized ultrasonic-assisted conditions, this extraction system provided a yield of 8.6 mg/g sophoricoside, representing approximately 27% higher efficiency than the conventional 70% ethanol control processed using identical parameters (6.78 mg/g). This relative superiority of the ChCl–Urea NADES over the hydroalcoholic solvent is strongly supported by parallel findings in the recent deep eutectic solvent extraction of Fructus Sophorae [24], wherein the authors’ choline chloride/ethylene glycol system likewise outperformed its own 70% ethanol control by a comparable margin. While our absolute extraction rates were slightly lower than those reported by Zuo et al. (2025) [24], these differences likely stemmed from variations in ultrasonic equipment and cavitation intensity rather than limitations of the NADES approach. Despite these discrepancies, both studies confirm that the tailored deep eutectic systems consistently outperform conventional organic solvents in improving the extraction of isoflavone glycosides. In addition, the screening of multiple NADES formulations at a baseline 30% water content identified ChCl–Urea (1:2), ChCl–Glycerol (1:2), and Glycerol–Urea (1:1) as the most promising compositions. Systematic single-factor and response-surface optimization further identified the maximal efficiency of Fructus Sophorae with 40% water incorporation. Water addition to NADES produces a biphasic effect on sophoricoside extraction. At a moderate water concentration, the yield could be increased due to reduced solvent viscosity, which enhances the diffusion into plant tissue and improves the analyte solubility [25,26]. However, further increase in water content results in decreased yields, as excess water destabilizes the solvent’s hydrogen-bonding network and diminishes solute–solvent interactions. This dual effect reflects the competing roles of water in simultaneously modulating both the physical properties and the chemical environment of the extraction medium [27]. These extraction outcomes highlight the potential of NADES-based green technologies for the efficient and eco-friendly utilization of herbal medicine resources.

An interesting consideration is the potential contribution of the NADES components to the bioactivity of the Fructus Sophorae extract. Unlike conventional organic solvents, e.g., ethanol, which are typically removed after extraction, the urea in the NADES may serve as a functional additive that enhances the bioactivity of Fructus Sophorae extract. Urea is a well-known keratolytic agent and penetration enhancer in dermatology [28]. Its ability to hydrate the skin and disrupt the localized hydrogen bonding of the stratum corneum is notable [28,29]. Consequently, the residual NADES may enhance sophoricoside transport into hair follicles. This suggests that the observed stimulation of hair growth and pigmentation is not solely dependent on the high concentration of sophoricoside, but rather the formation of a NADES–sophoricoside complex that improves solubility and bioavailability of the active ingredient(s). Future studies comparing pure sophoricoside with the NADES extract, as well as combination and interaction assays, will be required to determine whether the NADES components and sophoricoside act additively or interactively. By transitioning from a simple green extraction medium to a superior delivery system, this approach offers a distinct advantage over pure sophoricoside powder or traditional herbal tinctures, providing a stabilized, ready-to-use formulation with optimized potential for treating androgenetic alopecia and hair thinning.

In B16F10 cell culture, treatment with CU-40 was able to stimulate the promoter activities of pCRE-Luc and pMITF-Luc, as well as melanin synthesis, in a dose-dependent manner. This is the first evidence that the extract of Fructus Sophorae regulates melanogenesis via the cAMP/PKA/CREB–MITF axis in a physiologically relevant system. Consistent with the in vitro data, CU-40 promoted hair shaft elongation in ex vivo mouse vibrissae follicles (>60% increase after 72 h of treatment), outperforming the sFRP-1 inhibitor WAY 316606 (~25%). Taken together, the higher sophoricoside yield from ChCl–Urea NADES, together with its dual activation of the Wnt/β-catenin (growth) and cAMP/MITF (pigmentation) pathways, positions CU-40 as a promising green candidate for extraction of Fructus Sophorae to promote hair growth. Unlike conventional ethanol extraction, the NADES method provides better efficiency and sustainability while preserving biological activity [13,30]. These results offer a sustainable platform for utilizing Fructus Sophorae in natural hair care products, although further in vivo and human studies are needed to confirm efficacy and safety.

A critical consideration in interpreting our ex vivo findings is the biological distinction between the mouse vibrissae model and the human androgenetic alopecia scalp. While the vibrissae model does not reproduce androgen-dependent follicular miniaturization, the molecular pathway targeted by CU-40 is directly relevant to androgenetic alopecia pathogenesis. It is well-established that dihydrotestosterone suppresses Wnt signaling in dermal papilla cells derived from balding scalp, specifically through the inhibition of LEF/TCF-mediated transcriptional activity [31]. This suppression is a key mechanism driving follicular miniaturization. Our data demonstrate that CU-40 activates this very pathway, inducing GSK3β phosphorylation and upregulating downstream targets including β-catenin, AXIN2, and LEF-1. Therefore, while our model validates a general hair growth-promoting effect, the activation of Wnt/β-catenin signaling by CU-40 suggests a potential to counteract the specific signaling defect central to AGA, warranting further investigation in androgen-responsive or human scalp follicle models.

## 4. Materials and Methods

### 4.1. Cell Cultures and Materials

Murine melanoma B16F10 cells (Cat. No.: CRL-6475) were purchased from ATCC (Manassas, VA, USA), and an immortalized human dermal papilla cell line (DPC) (Cat. No.: T0501) was obtained from Applied Biological Materials (Richmond, BC, Canada). Both cell lines were cultured in DMEM medium supplemented with 10% FBS, 100 U/mL penicillin, and 100 μg/mL streptomycin. Direct co-cultures were prepared by seeding DPC and B16F10 cells at a 1:1 ratio. All cultures were maintained at 37 °C in a humidified atmosphere containing 5% CO_2_. Culture reagents were purchased from Thermo Fisher Scientific (Waltham, MA, USA). Sophoricoside (purity ≥98%) was sourced from Chengdu Herbpurify Ltd. (Chengdu, China) and dissolved in dimethyl sulfoxide (DMSO) to generate a 50 mM stock solution.

### 4.2. Animals

The animals were sourced from the Animal Care Facility at the Hong Kong University of Science and Technology (HKUST) and were cared for according to the standards established by the Department of Health of the Hong Kong SAR Government. All experimental procedures underwent a thorough review and were granted approval by the university’s Animal Ethics Committee (reference number: (22–26) in DH/HT&A/8/2/2 Pt.4). Housing conditions were kept constant at a temperature of 21 °C and a humidity level of 60%. The animals were kept on a fixed 12/12 h light/dark cycle and had free access to food and water at all times.

### 4.3. Preparation of NADES Extracts

Natural deep eutectic solvents (NADESs) were prepared by heating stoichiometric ratios of a hydrogen bond acceptor (HBA) and a hydrogen bond donor (HBD) with deionized water at 70–80 °C with constant stirring until a homogeneous mixture was obtained. To extract the bioactive metabolites, powdered Fructus Sophorae was separately suspended in each NADES. Both suspensions were then subjected to ultrasound-assisted extraction under identical, controlled conditions: a frequency of 40 kHz for 30 min at a steady temperature of 25 °C. Following centrifugation, the resulting supernatants were filtered through a 0.22 µm membrane and stored at −20 °C for subsequent analytical and bioassay procedures.

### 4.4. Raw Material and HPLC Condition

Fructus Sophorae raw material was collected from Shaanxi Province in 2023 and deposited at the Centre for Chinese Medicine, with authentication performed by Dr. Tina Dong. Its identity was verified according to the Hong Kong Materia Medica Standards. The raw material was then sonicated for 30 min with various formulations, including NADES, water, or ethanol, and this process was repeated twice. Solid debris was subsequently removed from the solvent by centrifugation and filtration. After filtration, the extracts were used directly in subsequent experiments at the indicated concentrations (% *v*/*v*). Separation by high-performance liquid chromatography (HPLC) with ultraviolet detection was performed on an Agilent HPLC 1200 series system (Agilent, Waldbronn, Germany). Fructus Sophorae extracts were separated on an ACE 5 C18 HPLC column (250 mm × 4.60 mm, 5 μm particle size). The mobile phase comprised 0.4% formic acid in water (solvent A) and a 50:50 mixture of acetonitrile and methanol (solvent B). The gradient program ran from 0 to 90 min, with a linear gradient of 85–70% solvent A. A 5 min pre-equilibration period was applied. The flow rate was 1 mL/min, the column temperature was maintained at 25 °C, and the injection volume was 10 μL. UV absorbance was measured at 266 nm.

### 4.5. Cell Proliferation Assay

Cell proliferation was evaluated by means of an MTT assay. DPCs were plated in 96-well plates at 5000 cells per well and incubated for 24 h. Following this initial incubation, cells were treated with Fructus Sophorae extracts for a further 24 h. The culture medium was then removed, and 100 μL of MTT solution was added to each well to reach a final concentration of 0.5 mg/mL, after which the plates were incubated at 37 °C for 1 h. At the end of this incubation, the medium was aspirated and substituted with 100 μL of DMSO, and the plates were agitated for 15 min. Absorbance was subsequently recorded at 570 nm.

### 4.6. DNA Transfection and Luciferase Reporter Assay

The TCF/LEF firefly luciferase reporter plasmid (pTOPflash) (Cat# 21-170), comprising two repeats that each contain three copies of the TCF binding site upstream of a thymidine kinase minimal promoter, was purchased from Upstate Biotechnology (Erie, PA, USA). The pMITF-Luc DNA constructs were supplied by Dr. Jacky Bonaventure (Institute Curie, PSL Research University, Paris, France), whereas the pCRE-Luc plasmid was obtained from BD Biosciences (Franklin Lakes, NJ, USA). As a positive control, cells were treated with Bt_2_-cAMP at a final concentration of 1 mM. Transfection of the various plasmids into DPCs or B16F10 cells was performed with Avalanche^®^ Transfection Reagent (EZ Biosystems, College Park, MD, USA) in accordance with the manufacturer’s instructions. Specifically, plasmids were introduced into DPCs or B16F10 cells by incubating them with the transfection mixture for 4 h, after which the medium was replaced with fresh medium, and the cells were maintained for 16 h prior to treatment. Following 24 h of treatment, the cells were harvested, and a luciferase reporter assay was conducted using a Pierce™ firefly luciferase glow assay kit (Thermo Scientific, Rockford, IL, USA) according to the manufacturer’s protocol. The resulting luminescence was quantified on a GloMax^®^ 96 Microplate Luminometer (Promega, Madison, WI, USA), and the measured values were normalized to the total protein content of each sample. CU-40 and Bt_2_-cAMP were assayed within the same experimental run and normalized to the same untreated control.

### 4.7. Real-Time PCR

Total RNA was extracted from cultured DPCs using RNAzol™ Reagent (Sigma-Aldrich, St. Louis, MO, USA). Thereafter, 2 μg of the extracted RNA was reverse transcribed with a PrimeScript™ RT reagent kit (Takara, San Jose, CA, USA) following the provided instructions. Template cDNAs were subjected to RT-PCR using the following specific primers: ALP(5′-AGC ACT CCC ACT TCA TCT GG-3′ and 5′-TGT CTT CCG AGG AGG TCA AG-3′); LEF-1 (5′-TTC CTT GGT GAA CGA GTC TG-3′ and 5′-GTG TTC TCT GGC CTT GTC GT-3′); AXIN2 (5′-GGG AGA AAT GCG TGG ATA C-3′ and 5′-CTG CTT GGA GAC AAT GCT GT-3′); IGF-1 (5′-TCA GAA GGG TAG CCC CTA GCT-3′ and 5′-TCA AGC CTG GGT ACT TTT AAC CA-3′); β-catenin (5′-CCC ACT AAT GTC CAG CGT TT-3′ and 5′-AAC CAA GCA TTT TCA CCA GG-3′); and glyceraldehyde 3-phosphate dehydrogenase (GAPDH) (5′-ACC TGA CCT GCC GTC TAG AA-3′ and 5′-TCC ACC ACC CTG TTG CTG TA-3′). Here, GAPDH, a housekeeping gene, was used as an internal control. Real-time PCR was performed with KAPA SYBR FAST qPCR kits and a LightCycler 480 instrument (Roche Molecular Biochemical, Indianapolis, IN, USA), according to the manufacturer’s protocol. Relative mRNA expression levels were determined using the 2^−ΔΔCt^ method.

### 4.8. SDS-PAGE and Western Blot Analysis

Cells were lysed in whole cell lysis buffer and shaken for 30 min at 4 °C, followed by centrifugation at 12,000× *g* at 4 °C for 10 min. The supernatants were collected, and protein concentrations were determined using a Bradford protein assay (Bio-Rad Laboratories, Hercules, CA, USA). The aliquots normalized to 40 μg of protein were applied to 8% sodium dodecyl sulfate (SDS)-polyacrylamide gels and then transferred to nitrocellulose membranes. The membranes were blocked with 5% BSA in Tris-buffered saline with 0.1% Tween-20 for 2 h. After blocking, the membranes were incubated at 4 °C overnight with specific primary antibodies, including anti-P-GSK3β at 1:1000 (#9336, Cell Signalling Technology, Danvers, MA, USA), anti-GSK3β at 1:1000 (SC-377213, Santa Cruz Biotechnology, Dallas, TX, USA), anti-P-CREB at 1:1000 (87G3, Cell Signalling Technology), and anti-CREB at 1:1000 (48H2, Cell Signalling Technology), followed by incubation with horseradish peroxidase-conjugated secondary antibodies (Sigma-Aldrich) at 25 °C for 1 h. The immune-reactive proteins were detected using an enhanced chemiluminescence (ECL) Western blot detection kit (Thermo Fisher Scientific). The intensities of the bands were quantified using a ChemiDoc Imaging System (Bio-Rad Laboratories). The intensities of protein bands were in the non-saturating range of calibration curves.

### 4.9. Measurement of the Amount of Melanin

For intracellular melanin measurement from a cell pellet, 100 μL of 1 N NaOH containing 10% DMSO was added to the pellet, which was then heated at 80 °C for 90 min. Absorbance was subsequently measured at 490 nm using an EnSpire multimode plate reader (Perkin Elmer, Waltham, MA, USA). Cells were treated with Bt_2_-cAMP at a final concentration of 1 mM as a positive control. CU-40 and Bt_2_-cAMP were assayed in the same experimental run and normalized to the same untreated control.

### 4.10. Ex Vivo Mouse Vibrissae Hair Follicle Culture

Four male C57BL/6 mice, aged 4 weeks, were euthanized by cervical dislocation. Their upper lip pad vibrissae hair follicles in the anagen phase were carefully extracted using microdissection techniques. These hair follicles were subsequently cultured in Williams E medium (Sigma-Aldrich, St. Louis, MO, USA) under controlled conditions at 37 °C and 5% CO_2_. For each experimental run, follicles obtained from a single mouse were allocated to individual treatment groups, with each group containing five hair follicles. The follicles were exposed to different treatments and incubated for 3 days. Throughout the incubation period, the length of the hair shaft was measured from day 0 to day 3.

### 4.11. Statistical Analysis

Statistical analysis was carried out using GraphPad Prism version 8.0 to compare data and determine statistical significance. Before conducting the analysis, all datasets were tested for normality using the Kolmogorov–Smirnov test and for equality of variances using the Brown–Forsythe test. Since the assumptions for parametric testing were met, the data were analyzed using one-way analysis of variance (ANOVA), followed by Tukey’s honestly significant difference post hoc test to account for multiple comparisons. The thresholds for statistical significance (*p*-values) were defined as follows: * *p* < 0.05, ** *p* < 0.01, and *** *p* < 0.001.

## 5. Conclusions

CU-40, a natural deep eutectic solvent composed of choline chloride and urea (1:2) with 40% water content can efficiently recovers sophoricoside from Fructus Sophorae. This extract activates Wnt/β-catenin signalling, upregulates downstream targets including β-catenin, AXIN2, LEF-1, ALP, and IGF-1, and induces GSK3β phosphorylation in dermal papilla cells. In B16 melanoma, the extract enhances MITF promoter activity and increases melanin production. Notably, in ex vivo mouse vibrissae hair follicle culture, the extract promotes hair follicle growth. These findings establish CU-40 as a sustainable and efficient NADES solvent for Fructus Sophorae that yields a bioactive extract promoting hair growth and pigmentation via the Wnt/β-catenin and cAMP/MITF pathways. Collectively, these results offer strong supporting evidence in favour of Fructus Sophorae as a possible candidate for drug development in the treatment of androgenetic alopecia, and provide interesting new avenues for applying Fructus Sophorae as a green herbal extract in combating hair loss. 

## Data Availability

The additional data supporting the manuscript are available from the corresponding author upon request.

## Supplementary Materials

The following supporting information can be downloaded at: https://www.mdpi.com/article/10.3390/ijms27198781/s1.

## Abbreviations

The following abbreviations are used in this manuscript:

ALP	alkaline phosphatase
AXIN2	axin-related protein 2
Bt_2_-cAMP	dibutyryl cyclic adenosine monophosphate
C91	CHIR99021
cAMP	cyclic adenosine monophosphate
ChCl	choline chloride
CRE	cAMP response element
DMSO	dimethyl sulfoxide
DPC	dermal papilla cell
GSK-3β	glycogen synthase kinase 3 beta
HBA	hydrogen bond acceptor
HBD	hydrogen bond donor
IGF-1	insulin-like growth factor 1
LEF-1	lymphoid enhancer-binding factor 1
MITF	microphthalmia-associated transcription factor
NADES	natural deep eutectic solvents
sFRP-1	secreted frizzled-related protein 1
TCF/LEF	T-cell factor/lymphoid enhancer factor
