# Supplementary material for: Fructus Sophorae Extract Generated Using the Natural Deep Eutectic Solvent ChCl Urea Activates Wnt/β Catenin and cAMP/MITF Signaling to Stimulate Hair Growth and Pigmentation
Source: Int J Mol Sci. 2026 Sep 30;27(19):8781. doi: 10.3390/ijms27198781 (PMC13664810; doi:10.3390/ijms27198781)
Supplement: Supplementary file 1 [file ijms-27-08781-s001.zip › ijms-4551394-supplementary.pdf]

	Molar ratio	SOP (mg/g)
ChCl-Urea	1:2	4.98
Glycerol-urea	1:1	4.46
ChCl-Glycerol	1:2	4.31
ChCl-Glucose	1:1	1.67
ChCl-Ascorbic Acid	2:1	1.31
Glycerol-Lactic Acid	1:1	1.19
ChCl-Sorbitol	1:1	1.08
ChCl-Citric Acid	1:2	0.85
Glycerol-Citric Acid	1:1	0.72
Sorbitol-Citric Acid	1:1	0.53
Water		3.33
70% ethanol		7.44
100% ethanol		3.67

Supplementary Figure S1. Screening of ten NADES formulations and their optimal molar ratios.

Sophoricoside extraction results for all ten natural deep eutectic solvent (NADES) formulations, with the optimal molar ratio (HBA:HBD) indicated for each formulation.

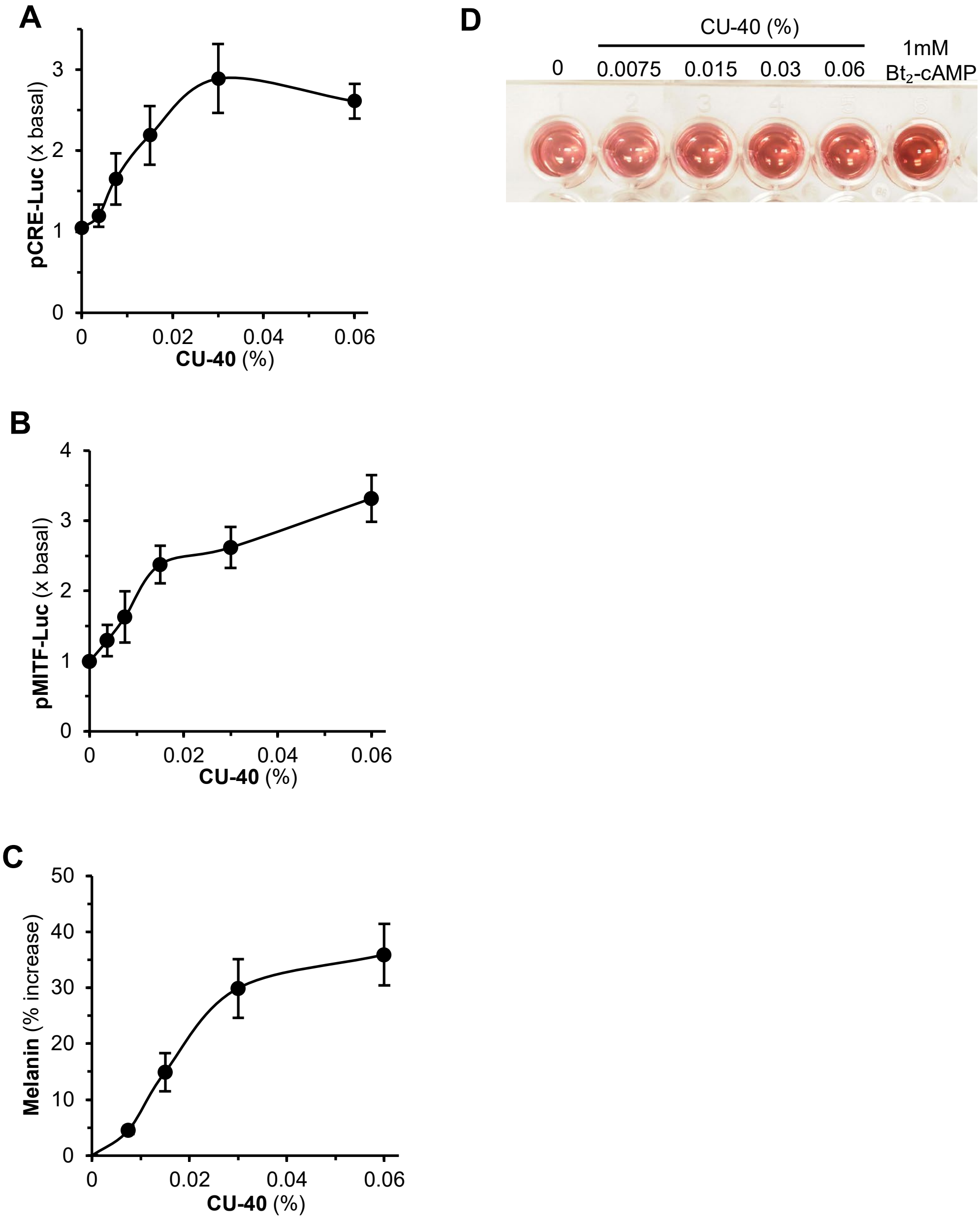

Supplementary Figure S2. CU-40 promotes cAMP/MITF-dependent melanogenesis in B16F10 – DPC co-culture.

(A) The luciferase activity assay of pCRE-Luc-transfected B16F10 cells were co-cultured with DPCs (ratio 1: 1) and treated with different concentrations of CU-40 (as indicated) for 24 hours. **(B)** The luciferase activity assay of pMITF-Luc-transfected B16F10 cells co-cultured with DPC as in (A). **(C)** The treatment was as in (A). The melanin content measured by spectrophotometry at 405 nm and normalized to total protein in the co-cultures. Different concentrations of CU-40 were employed (as indicated). **(D)** Representative photographs of DMSO-dissolved cell pellets from each treatment group in a 6-well plate, showing color intensity consistent with the quantified melanin content in Fig.

5E. Data are normalized and expressed as fold of change (x basal) or % of increase to control, in mean ± SEM, $n = 4$. * $p < 0.05$, ** $p < 0.01$, *** $p < 0.001$.
